# Subthalamic Nucleus Deep Brain Stimulation Does Not Improve Visuo-Motor Impairment in Parkinson’s Disease

**DOI:** 10.1371/journal.pone.0065270

**Published:** 2013-06-11

**Authors:** Simon D. Israeli-Korn, Shraga Hocherman, Sharon Hassin-Baer, Oren S. Cohen, Rivka Inzelberg

**Affiliations:** 1 Parkinson’s Disease and Movement Disorders Clinic, Sagol Neuroscience Center and Department of Neurology, Sheba Medical Center, Tel Hashomer, Israel; 2 Department of Physiology, Faculty of Medicine, Technion, Haifa, Israel; 3 Sackler Faculty of Medicine, Tel Aviv University, Tel Aviv, Israel; Oslo University Hospital, Norway

## Abstract

**Objective:**

To evaluate how bilateral subthalamic nucleus deep brain stimulation (STN-DBS) affects visuo-motor coordination (VMC) in patients with Parkinson’s disease (PD).

**Background:**

VMC involves multi-sensory integration, motor planning, executive function and attention. VMC deficits are well-described in PD. STN-DBS conveys marked motor benefit in PD, but pyscho-cognitive complications are recognized and the effect on VMC is not known.

**Methods:**

Thirteen PD patients with bilateral STN-DBS underwent neurological, cognitive, and mood assessment before VMC testing with optimal DBS stimulation parameters (‘on-stimulation’) and then, on the same day without any medication changes, after DBS silencing and establishing motor function deterioration (‘off-stimulation’). Twelve age-matched healthy controls performed 2 successive VMC testing sessions, with a break of similar duration to that of the PD group. The computer cursor was controlled with a dome-shaped ‘mouse’ hidden from view that minimized tremor effects. Movement duration, hand velocity, tracking continuity, directional control variables, and feedback utilization variables were measured. MANOVA was performed on (1) clinically measured motor function, (2) VMC performance and (3) mood and attention, looking for main and interaction effects of: (1) group (controls/PD), (2) test-order (controls: first/second, PD: on-stimulation/off-stimulation), (3) path (sine/square/circle) and (4) hand (dominant/non-dominant).

**Results:**

Unified PD Rating Scale (UPDRS) Part III worsened off-stimulation versus on-stimulation (mean: 42.3 versus 21.6, p = 0.02), as did finger tapping (p = 0.02), posture-gait (p = 0.01), upper limb function (p<0.001) and backwards digit span (p = 0.02). Stimulation state did not affect mood. PD patients performed worse in non-velocity related VMC variables than controls (F_(5,18)_ = 8.5, p<0.001). In the control group there were significant main effects of hand (dominant/non-dominant), path (sine/square/circle) and test-order (Test_1/Test_2). In the PD group, hand and path effects, but no test-order (on-stimulation/off-stimulation), were found.

**Conclusions:**

‘Low-level’ clinically-measured motor function responds to STN-DBS but ‘high-level’ motor and cognitive functions relating to VMC may be unresponsive to STN-DBS.

## Introduction

Parkinson’s disease (PD) is a motor disorder characterized by rigidity, bradykinesia and tremor as well as cognitive and executive deficits even in the early stages of the disease [1]. Patients with PD have impaired visuo-motor coordination (VMC) [2]–[5] as well as ‘high-level’ functions that VMC may rely on including motor sequencing [6], executive function [7], [8], motor planning [6], [9]–[11], sensory integration [12]–[14], visuo-spatial function [15], visuo-motor integration [15] and attention [16], [17]. Progressive VMC impairment is associated with PD and may accompany or even precede the early clinical motor hallmarks of the disease [5], [18]. Cognitive dysfunction has been reported in all stages of PD [19]. Between 30 and 70% of non-demented patients with advanced PD have frontal dysfunction [20] resulting in impaired function work and at home [21] and a lower perceived quality of life [22].

Bilateral deep brain stimulation of the subthalamic nucleus (STN-DBS) improves rigidity, akinesia and tremor [23]–[25], allows reduction of the dose of dopaminergic replacement therapy and improves levodopa-induced involuntary movements [26]–[28]. Furthermore, benefits are seen in patients’ functional independence over the long-term [29], [30] and their quality of life over the short-term (as shown in a recent study in early PD [31]).

On the other hand gait and posture control, shown to involve high-level functions in PD such as attention and executive function [32]–[34] and visuo-spatial function [35], appears to deteriorate in STN-DBS treated PD patients at the same or an accelerated rate compared to PD patients without STN-DBS [30], [36]–[38]. Furthermore, bilateral STN-DBS may produce behavioral and cognitive symptoms both acutely and chronically. Chronically, neuro-psychiatric and cognitive morbidity that has been reported with long-term STN-DBS treatment includes executive dysfunction [39], [40], delayed free recall [41], impaired verbal fluency [40], [42]–[45] and depression [46], [47]. The pre-supplementary motor area and anterior cingulate in the frontal cortex are considered to be involved in the high-level organization and planning of motor activity. Regional cerebral blood flow in these areas, as measured by single-photon emission computed tomography (SPECT), has been shown to increase with STN-DBS and to correlate with clinical motor improvement [48]. Despite the wide spectrum of motor and non-motor manifestations of PD, routine assessment of patients with PD in the context of optimizing pharmacological and DBS therapy is largely reliant on the Unified Parkinson’s Disease Rating Scale (UPDRS) clinical rating scale [49], which despite well-established inter-rater reliability [50] may be limited by its sensitivity to the placebo effect [51], poor measurement of hypokinesia [52], as well as other factors [53]. There is a need to establish user-friendly, valid and reliable quantitative evaluative methods for assessing both motor and cognitive functional outcome measures in PD. VMC testing as described herein, as well as in previous publications [54]–[56], offers a tool for quantitatively evaluating motor function. The complexity of the tasks and the abundance of performance measures allow analysis of movement speed, directional control, motor planning, motor sequencing, executive function, sensory integration, attention, on-line error correction and visuo-motor integration, all of which are impaired in PD (see above).

Although the patient’s motor disability is known to improve with STN-DBS, it is not clear whether and which high-level motor functions improve or deteriorate. Since VMC in patients with PD is largely independent of the motor disability of the executing limb yet correlates with impaired axial control [56] and axial control is unaffected or worsened by STN-DBS [30], [36]–[38], we hypothesized that there would be a divergent effect of STN-DBS on clinical measures of motor function versus visuo-motor performance.

Thus, in the present study we aimed to quantify the effect of STN-DBS on certain measures of visuo-motor function that reflect both high and low-level processes and thereby gain understanding on the mechanisms of STN-DBS and why high-level motor function may fail to improve or even deteriorate with STN-DBS treatment. Furthermore, we aimed to demonstrate a potential application for a computer-assisted tool for the quantitative assessment of complex motor functions to supplement bed-side clinical rating scales.

## Methods

### Ethics Statement

Approval according to the Declaration of Helsinki was received from the institutional review board of the Sheba Medical Center prior to the commencement of the research. All subjects gave fully informed written consent prior to their participation in this study.

### Subjects

Subjects with PD were recruited from the Parkinson’s Disease and Movement Disorders clinic at the Sheba Medical Center. All subjects were post STN-DBS. Inclusion criteria were (1) a diagnosis of PD treated with bilateral STN-DBS, (2) a delay of at least 3 months between STN-DBS insertion and VMC testing, (3) the absence of any post-operative complications and (4) stable DBS stimulation parameters and a stable anti-parkinsonian medication regime for at least one month prior to testing. Exclusion criteria were major psychiatric symptomatology and dementia [57].

Age-matched healthy subjects (as defined by the absence of any neurological or psychiatric history) were recruited on a voluntary basis from staff and acquaintances of staff in the Neurology Department of the same institution.

### Study Design

On the day of testing (starting between 9 and 10 am), each subject underwent a neuro-cognitive assessment followed by two sessions of VMC testing, first in the ‘on-stimulation’ state (with optimal stimulation parameters) and then in the ‘off-stimulation’ state (after silencing the implantable pulse generator). In both states regular anti-parkinsonian medications were continued without any alterations to the dosage or the timing. The onset of the off-stimulation state was determined clinically after a delay of 28±6 minutes (no less than 20) from turning off the implantable pulse generator. Before each session of VMC testing, tests of attention, mood, clinical motor function and gait were performed. On the basis of our previous experience (unpublished) we expected the magnitude of the learning effect on VMC performance to be small and possibly cancelled out by fatigue-related effects. Furthermore, we expected stimulation sensitive VMC variables to deteriorate to a similar extent and at a similar rate to clinical measures of motor function. Therefore, we assumed that switching off the DBS and waiting for a significant deterioration of the clinical motor signs would lay the stage for a similar deterioration of stimulation sensitive VMC measures. With this in mind, the order of testing was pre-determined to be on-stimulation followed by off-stimulation.

In order to estimate the effect of learning on VMC testing performance a healthy age-matched control group underwent two consecutive VMC testing sessions with a break lasting 53±23 minutes (mean ± SD) which was of similar duration between the two sessions, p = 0.08. All subjects performed two practice tasks prior to being tested in order to understand the experimental procedure and to reduce any possible learning effects.

The duration of each VMC testing session was 18±7 minutes for patients and 9±1 minutes for controls. Altogether the duration of testing for patients was 133±42 minutes (mean ± SD).

#### Cognitive testing

Cognitive testing included the following examinations: Mini-Mental State Examination (MMSE) [58], Beck Depression Inventory (BDI) [59], phonemic and semantic verbal fluency (previously validated in Hebrew speakers for the letters ‘*Bet*’, ‘*Gimmel*’ and ‘*Shin*’ and for the categories ‘Animals’, ‘Fruits and Vegetables’ and ‘Vehicles’) [60] and the Frontal Assessment Battery (FAB) [61]. All subjects were proficient in Hebrew and all tests were performed in Hebrew.

#### Clinical motor function, mood and attention testing

Prior to each VMC testing session subjects underwent assessments of (1) clinical motor function (described below), (2) mood (using the Visual Analog Mood Scale a visual self-rating scale) [62]–[64] and (3) attention (digit span forwards and backwards) [65].

Clinical motor function was tested using the UPDRS Part III, timed-up-and-go (time taken to arise from a chair with no arms, walk 3 meters then return to the chair and sit) [66] and a finger tapping test whereby subjects tapped an electronic tapper (Western Psychological Services, Los Angeles, California) with their index finger as quickly as possible for 10 seconds [67].

#### VMC testing

**Figure 1:**
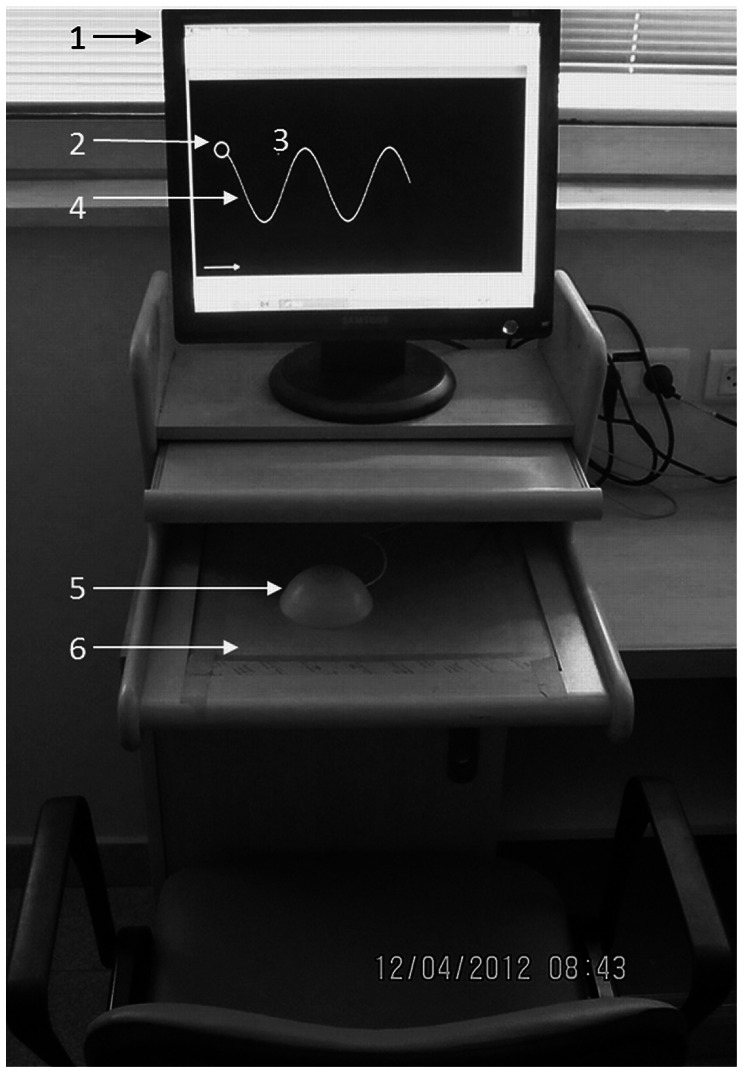
Experimental apparatus. (1) Computer monitor. (2) Circular target indicating the start of the experiment. (3) Cursor. (4) Path of the task: in this case sinusoidal but may also be square or circular. (5) Dome-shaped unseen ‘mouse’ that cancels the effect of tremor. (6) Digitizing tablet.

The subject sat comfortably in front of a computer monitor, each hand was tested separately. The tested hand rested on a specially-designed dome-shaped ‘mouse’ hidden from view that controlled the cursor position on the monitor by its movements in the horizontal plane over the low-friction surface of a digitizing tablet (Figure 1). Movement of the ‘mouse’ was represented in a ‘one-to-one’ fashion by the cursor in terms of distance, direction and velocity of movement. The monitor displayed the 1-pixel sized cursor and either a sinusoidal, square or circular path. Each path was 1-pixel thick and extended over a distance of 32 cm. In the case of a ‘tracking’ task (Figure 1) a moving circular target of 1 cm diameter was also displayed and the subject was instructed to maintain the cursor within the target and as close to target center as possible. The target was initially positioned at one end of the path and started to move once the cursor was brought within its boundaries. The target then moved at a pre-determined, constant speed of 18 mm/sec for the square and circular paths and a variable speed for the sinusoidal path (16 mm/sec at the peaks and troughs with highest curvature and 20 mm/sec in the middle, straight sections). If the cursor strayed outside the moving target, the target would cease to progress along its path until the cursor was moved back into it. This was considered a ‘tracking interruption’. In the case of tracing tasks the target, serving to indicate the starting point of the path, disappeared once the subject brought the cursor into it. The subject then traced the path with the cursor at his/her own comfortable pace. One of the main differences between the tracing and tracking tasks is the degree of internal versus external cueing for the purpose of directional control. Given that patients with PD are well-known to have more pronounced impairment of motor function as demands for internal cueing increase [5], [68]–[70], the utilization of the two types of tasks provided an extended range of functional testing as relevant to PD.

The following 8 variables were used to measure VMC testing performance: (1) time spent outside the target during tracking (OutT_k), (2) number of times that the cursor moved outside of the target, i.e. number of tracking interruptions (NI_k), (3) mean distance (mm) of the instantaneous cursor position from target center (D_k), (4) mean absolute difference between the target speed and the cursor speed (SE_k), (5) mean distance between trajectory of the cursor and the model path (ME_c), (6) mean component of the movement vector perpendicular to the path (directional error) expressed as a percentage of the total movement vector (DE_c), (7) cumulative time during which directional error exceeded half the maximal possible level (TF_c) and (8) mean cursor velocity (V_c). The first 4 variables were derived from tracking tasks and the last 4 from tracing tasks, as indicated by the suffices ‘_k’ and ‘_c’ respectively. The first two variables (OutT_k and NI_k) relate to tracking continuity, variables 6 and 7 (DE_c and TF_c) relate to direction control, variable 4 (SE_k) relates to speed control and variables 3 and 5 (D_k and ME_c) relate to visual feedback.

#### Data analysis

The UPDRS Part III scores were pooled into the following sub-categories: (1) upper limb function (sum of items 22–25 inclusive: rigidity, finger taps, hand movements and rapid alternating movements respectively, for the upper limbs), (2) upper limb tremor (item 20: resting tremor, for the upper limbs) and (3) posture and gait (sum of items 28, 29 and 30: posture, gait and postural stability respectively).

Exploratory statistics to determine whether the variables met the assumption of being normally distributed and the degree to which the variables within each ‘variable cluster’ (clinical motor function, mood, attention and VMC) correlated with each other were performed using the Shapiro-Wilk Test and two-tailed Pearson’s correlation tests, using a significance level of 0.05. A separate MANOVA was performed on each variable cluster looking for main and interaction effects of: (1) *group* (controls/PD), (2) *test-order* (controls: first/second, PD: on-stimulation/off-stimulation), (3) *path* (sine/square/circle) and (4) *hand* (dominant/non-dominant). For the data to be appropriate for MANOVA, dependent variables should be moderately correlated with each other (defined by correlation coefficients within the range of 0.2 to 0.6) [71]. Variables were excluded from further analysis or analyzed separately if the absolute value of their correlation coefficients exceeded or were below this moderate range. For the purposes of correlation and descriptive statistics, data for variables were taken across all subjects from both groups (n = 24) and across all 12 within-subject conditions (*test-order*: 2; *hand*: 2; *path*: 3). Statistical analyses were performed using IBM® SPSS®, Statistics 19.

## Results

### Subject Recruitment and Drop-out (Figure 2)

Thirty-one patients with PD post STN-DBS were approached, 5 were excluded because of dyskinesias, unstable DBS parameters, cognitive problems or severe akinesia in the off-stimulation state and 8 patients refused to participate. After testing in the on-stimulation state 5 patients refused to be tested in the off-stimulation state. Of the 13 patients who underwent the full protocol of being tested in both stimulation conditions, one was unable to complete some of the tasks in the off-stimulation state.

**Figure 2:**
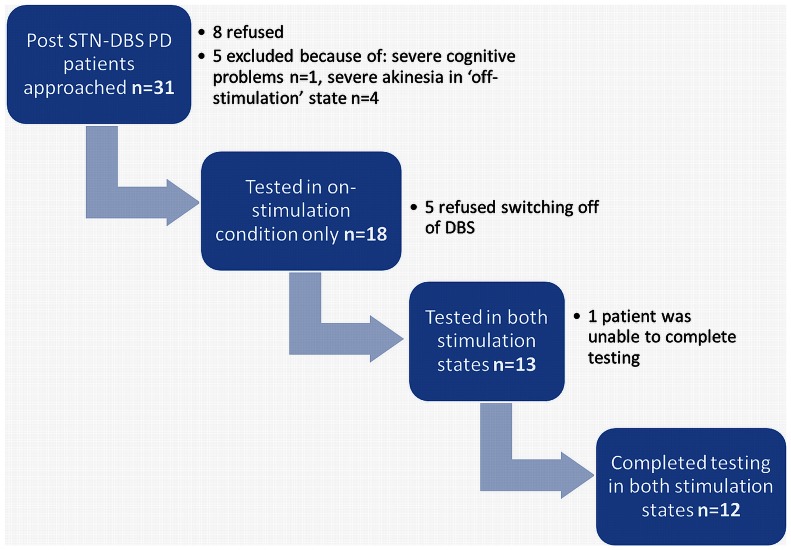
Flow chart for patient recruitment.

### Patient Demographics and Cognitive Tests (Table 1)

Of the 13 subjects (12 males) who underwent testing in both stimulation states, 11 were right-handed, all had asymmetrical bilateral disease prior to bilateral STN-DBS and 5 were more affected on their dominant side (with respect to handedness). Patients were 61.8±7.7 years old, and disease duration was 12.6±4.4 years (mean ± SD). The mean Hoehn and Yahr disease stage was 3 (range: 2 to 3) both on-stimulation and off-stimulation. PD patients were tested 2.5±1.4 years post STN-DBS insertion (mean ± SD).

**Table 1:** Patient demographics and cognitive tests.

										MMSE	FAB	MMSE	FAB				
Subject initials	Gender	Age	DiseaseDuration	Monthspost-op	Handed-ness	Most affected side	Pre-op^1^ LDED^2^(mg)	Post-op LDED^2^(mg)	BDI	Pre-operative^1^		Post-operative		PVF	SVF	PVFc^3^	SVFc^3^
BS	M	61	11	8	R	L	1248	599	10	28	15	30	17	22	42	14.5	12.5
CM	M	56	9	21	R	L	1090	1039	3	NA	18	29	18	38	51	−1.5	3.5
DI	M	61	8	6	R	L	1348	266	8	30	17	28	15	22	23	14.5	31.5
DK	M	73	17	43	L	L	1500	403	4	NA	NA	28	15	26	27	10.5	27.5
ES	M	46	6	8	R	R	2700	920	36	30	18	29	11	10	31	20.8	16.6
IP	M	64	15	52	R	L	1500	460	31	NA	NA	29	18	21	38	15.5	16.5
IS	M	61	11	38	R	R	1530	495	19	NA	NA	29	13	25	38	11.5	16.5
IV	M	70	15	51	R	R	2275	300	11	NA	NA	28	18	34	44	2.5	10.5
JK	M	52	8	31	R	L	1000	500	1	NA	NA	28	18	35	37	1.5	17.5
MA	M	59	18	49	L	R	1225	1000	14	NA	NA	25	6	11	18	25.5	36.5
YA	F	67	13	27	R	R	1268	200	24	28	17	25	5	1	0	29.8	47.6
YS	M	72	21	31	R	L	1090	375	10	24	16	29	17	21	38	15.5	16.5
ZA	M	62	12	29	R	L	1225	530	3	27	NA	30	18	33	39	3.5	15.5
Mean	-	61.8	12.6	30	-	-	1461	545	13.4	27.8	16.8	28.2	14.5	23.0	32.8	12.6	20.7
Std	-	7.7	4.4	16	-	-	492	276	11.1	2.2	1.2	1.6	4.6	10.8	13.2	9.4	11.9

1 Pre-operative data was derived from patients’ records.

2 LDED: levodopa equivalent dose.

3 Suffix ‘c’ indicates corrected score as calculated by: actual score subtracted from the expected score according to normative data for age group [60]. Thus a lower corrected score reflects better performance.

Please refer to the text (Methods, Study Design and Cognitive Testing) for definitions of abbreviations.

### Preparatory Correlation Analysis Prior to MANOVA

As described in the methods section Pearson’s correlation analysis was performed on all the variables to determine the degree of correlation and thus the appropriateness of subsequent MANOVA testing. Highly correlated variables were excluded from further analysis and poorly correlating variables were analyzed separately.

#### Clinical motor function

On Pearson’s correlation analysis the following variable pairs were found to be highly correlated: timed-up-and-go and posture-gait, and total UPDRS Part III and posture-gait (*r* values: 0.73 and 0.69 respectively, p<0.001 for both). Thus timed-up-and-go and total UPDRS Part III were disregarded from subsequent MANOVA analysis. Since the scores of lateralized variables of clinical motor function (finger tapping, upper limb function and upper limb tremor) were highly correlated between the dominant and non-dominant hands (with *r* values between 0.77 and 0.97, p<0.001 for all), the arithmetic mean of the scores of the two hands were used and the effect of hand was disregarded for statistical analysis of clinical motor function.

#### Mood

For mood variables the only variable pair with a correlation coefficient above the moderate range was tiredness and sadness (r = 0.70, p<0.001). Thus tiredness and sadness scores were combined using the arithmetic mean for each subject.

#### Attention

Attentional variables correlated poorly with mood variables and were thus analyzed separately.

#### VMC testing

Tracing velocity (V_c) correlated poorly with other VMC variables (absolute *r* values between 0.07 and 0.18) and thus was analyzed separately. Time spent outside the target (OutT_k) and time during which directional error exceeded half the maximal possible level (TF_c) correlated the most strongly with other VMC variables (with the arithmetic mean of all *r* values being 0.61 and 0.56 respectively, p<0.001 for all variables except tracing velocity), and were thus excluded from subsequent MANOVA analysis.

### MANOVA Analyses

#### Clinical motor function (Table 2)

There was a significant difference in overall clinical motor function between the two stimulation states as revealed by MANOVA, p = 0.02. Subsequent univariate tests revealed a significant effect of stimulation state on finger tapping: F_(1,12)_ = 6.6, p = 0.02; posture-gait: F_(1,12)_ = 9.45, p = 0.01 and upper limb function: F_(1,12)_ = 28.9, p<0.001, but no effect on upper limb tremor.

**Table 2:** Clinical motor function, attention and mood in PD patients.

		Test 1 (on-stimulation)	Test 2 (off-stimulation)	p-value
Variable group	Variable	Mean (standard error)		
Clinical motor function^1^	Finger tapping^2^	58.3 (1.0)	46.7 (4.5)	**0.02**
	Posture_gait^4^	3.00 (0.5)	4.62 (0.7)	**0.01**
	Upper limb function^2^	4.65 (0.6)	9.42 (0.9)	**<0.001**
	Upper limb tremor^2^	0.15 (0.1)	1.58 (0.9)	0.15
Mood^1,3^	Afraid	15.4 (4.0)	27.8 (8.3)	0.16
	Angry	22.0 (9.6)	29.9 (8.3)	0.48
	Confused	29.2 (7.4)	30.9 (7.8)	0.87
	Energetic	43.2 (7.4)	18.9 (4.4)	0.02
	Happy	41.7 (8.7)	26.9 (6.6)	0.06
	Sad_tired^5^	58.9 (9.0)	64.8 (8.8)	0.47
	Tense	15.3 (4.0)	27.8 (8.3)	0.86
Attention^1^	Digit span backwards	5.69 (0.7)	4.69 (0.6)	**0.02**
	Digit span forwards	8.31 (0.8)	8.85 (0.7)	0.19

1 Separate MANOVAs were performed for each variable group.

2 The mean value for both hands is used.

3 Significance level of 0.007 (Bonferroni correction for 7 non-VMC variables) [72].

4 The sum of UPDRS items 28–30.

5 Sad_tired is the arithmetic mean of the scores for sad and tired. The mean was taken because of the high correlation value (see text for explanation).

#### Mood (Table 2)

With respect to mood-related variables, no overall difference between the two stimulation states was seen on multivariate testing (p = 0.24) and on subsequent univariate testing no variables differed significantly.

#### Attention (Table 2)

There was a significant difference overall in the attentional variables between the two stimulation states as revealed by MANOVA, p = 0.05. Subsequent univariate testing revealed a significant stimulation effect on digit span backwards only: F_(1,12)_ = 6.5, p = 0.02.

#### VMC testing

VMC variables were analyzed by MANOVA testing the effects of: *group* (i.e. healthy controls, PD patients), *hand* (dominant, non-dominant), *path* (sine, square, circle) and *test-order* (Test 1, Test 2 for controls; on-stimulation, off-stimulation for PD).

There was a significant difference overall in the non-velocity VMC variables between PD patients and controls, p<0.001. Significant differences between PD patients and controls were seen for NI_k: 26.7 (PD) versus 3.42 (controls); D_k: 7.40 versus 3.78; ME_c; 48.8 versus 21.1 and SE_k: 3.67 versus 1.60, p≤0.02 (Table 3). Note that a larger score indicates worse performance. A separate factorial ANOVA on tracing velocity did not reveal a significant difference between PD patients and controls, with the mean scores for PD patients versus controls being 20.5 versus 20.6, p = 0.99.

**Table 3:** Effect of *group* and *test-order* on VMC variables.

	Controls			PD Patients			
	Test 1	Test 2	p *(test-order)*	Test1/On-stimulation	Test2/Off-stimulation	p *(test-order, stimulation)*	p *(group*)
NI_k	4.19	2.65	0.01	24.9	28.6	0.57	**<0.001**
D_k	3.92	3.63	**<0.01**	7.4	7.3	0.81	**<0.01**
ME_c	24.7	17.6	0.19	45.8	47.9	0.82	**0.02**
DE_c	27.8	25.7	**<0.01**	29.2	29.3	0.96	0.44
SE_k	1.79	1.39	0.04	3.6	3.9	0.73	**0.01**
V_c^1^	19.5	21.6	0.13	22.6	18.4	0.16	0.99
Overall			0.15			0.98	**<0.001**

Test 1 and Test 2 refer to the first and second testing sessions respectively. Note that a larger score for all these variables with the exception of V_c indicates worse performance. An adjusted significance level of 0.01 according to the Bonferroni correction for 5 non-VMC variables [72] was used. Please refer to the text (Methods, Study Design and VMC Testing) for definitions of abbreviations.

1 A higher score in this variable (in contrast to other variables) indicates an improvement.

There was no significant difference overall in the non-velocity VMC variables between Test 1 and Test 2 both in controls (p = 0.16) and in patients (p = 0.98). Similarly no difference was found in tracing velocity between Test 1 and Test 2 in either subject group (p = 0.13 for controls and p = 0.16 for patients, Table 3). On subsequent univariate testing, controls had a significantly improved performance on retesting in D_k (p<0.01) and DE_c (p<0.01), with an adjusted significance level of 0.01 (according to the Bonferroni correction for 5 variables) [72].

With respect to non-velocity VMC variables overall there was a significant effect of *path* in both controls: F_(10,38)_ = 11.2, p<0.001 and patients: F_(10,38)_ = 2.45, p = 0.02 but no effect of *hand* in either group. With respect to tracing velocity, there was a significant effect of *path*: F_(2,10)_ = 12.7, p<0.01, and *hand*: F_(1,11)_ = 14.9, p<0.01, in controls only. Results of univariate testing are presented in Table 4.

**Table 4:** Univariate testing of *hand* and *path* on VMC variables.

Group	Factor	Variable	Hypothesis DoF	Error DoF	F	p
PD	Path	ME_c	1.94	21.3	4.34	0.03
		DE_c	1.54	16.9	5.54	0.02
Controls	Hand^1^	SE_k	1	11	11.1	<0.01
		V_c	1	11	14.9	<0.01
	Path	NI_k	1.36	15.0	8.95	<0.01
		D_k	1.3	14.3	8.80	<0.01
		DE_c	1.97	21.2	29.8	<0.001
		SE_k	1.89	20.8	7.88	<0.01
		V_c	1.86	20.5	11.7	<0.01

1 Significance level of 0.01 (Bonferroni correction for 5 variables) [72]. Only findings of statistical significance are presented. Please refer to the text (Methods, Study Design and VMC Testing) for definitions of abbreviations.

## Discussion

Our study shows divergent effects of bilateral STN-DBS cessation on medicated PD patients. As expected, comparison of clinical motor function (posture-gait, upper limb function derived from the UPDRS Part III and electrically recorded finger tapping) all deteriorated significantly in the off-stimulation state, with a greater than two-fold increase in the clinical rating score of upper limb dysfunction on average. The magnitude of this effect is similar (and opposite in sign) to the improvement in UPDRS Part III scores following STN-DBS initiation post-operatively, as reported in the literature [73]. Recent findings that STN-DBS primarily modulates the influence of motor cortex neuronal activity on STN may explain this effect [74]. However there may be a gradient whereby high-level motor function improves to a lesser degree than simpler repetitive limb movements, as seen in the present study. This finding suggests that even within the motor system itself, the effects of DBS are more strongly manifested in simple aspects of hand/arm movements. Posture and gait control involves high-level cognitive processes in PD [32]–[35], is less responsive to STN-DBS as shown in this study, may progressively deteriorate post STN- DBS [30], [36]–[38] and correlates with VMC [56].

With respect to visuo-motor function, PD patients had markedly impaired visuo-motor function compared to controls (as measured by all VMC variables, with the exception of velocity and directional error). Neither velocity nor the non-velocity variables (on a multivariate level) were affected by *test-order* nor was there a test-order*group interaction, implying that no learning effect or stimulation effect was detected in either subject group. However, on univariate analysis, controls improved their scores in directional error and distance to target on retesting. If we assume that this learning effect also occurred in PD patients, and that fatigue-related deterioration in performance on retesting was negligible, then there may have been an STN-DBS related effect that improved VMC performance but was not detected in the present study. Even if this is the case, the very small presumed improvement of the patients’ VMC performance under DBS stands in sharp contrast to the large benefit in simple manual motor performance that follows subthalamic stimulation and that was detected in the present study. Moreover, the experimental design was sensitive to changes in performance between the other task conditions (*hand* and *path*) suggesting that there was adequate power to detect any *test-order* effects. Thus, our results suggest that DBS may improve VMC to a minimal extent at best.

The quality of tracking in PD has been shown to be largely influenced by cognitive-executive dysfunction, whereby the frequency of tracking interruptions is much higher in PD patients than in normal controls [75]. The present study shows no improvement in the frequency of tracking interruptions with DBS suggesting that this therapy does not affect the patient’s executive function.

**Figure 3:**
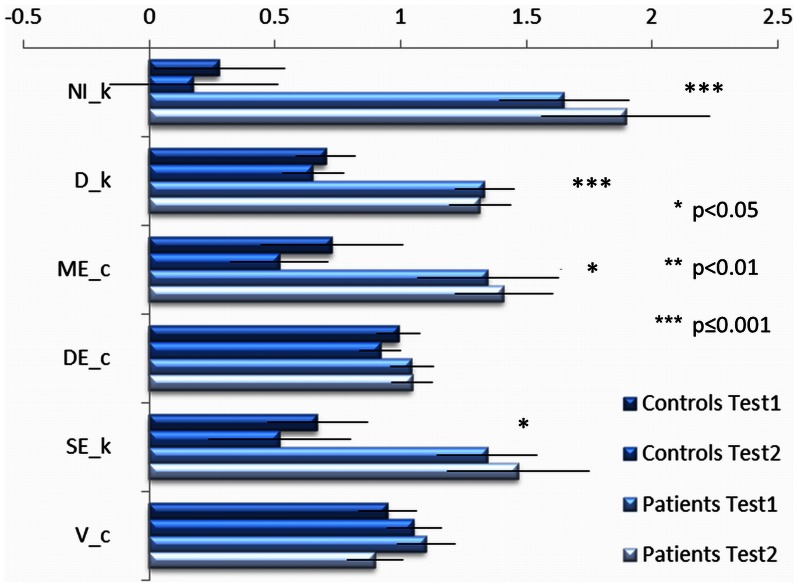
Normalized visuo-motor function in patients and controls. The data was normalized by dividing the mean scores for each group for each test (across all *hand* and *path* conditions) by the mean scores across both groups and tests for each variable. Thus x = 1 represents the mean value. The error bars represent the normalized 95% confidence interval (derived from estimated marginal means analysis in MANOVA, SPSS). P-values relate to the group differences. Please refer to the text (Methods, Study Design, VMC Testing) for definitions of abbreviations.

Significant differences between patients and age-matched controls have also demonstrated patients’ difficulty in controlling the distance to target center in the tracking tasks, as well as the distance between cursor trajectory and the model path in tracing tasks. This reduced ability to approximate the desired target/path must, in the absence of deficient directional control, hinge on a reduced ability to utilize visual feedback for the generation of corrective hand movements. Again both measures appear to be invariant to subthalamic stimulation (Figure 3). Since the incorporation of visual feedback in visuo-motor planning is dependent on extensive integration of activity in different parietal and frontal cortical fields, it is more likely to be associated with high-level control than with the production of simple movements. VMC may therefore represent another dimension in the realm of functions which are not responsive to subthalamic stimulation. Further experiments are needed to investigate neural correlates of visuo-motor function and STN-DBS to determine whether areas involved in visuo-motor function are less affected by STN-DBS.

With respect to movement velocity, it was found not to differ significantly between PD patients and controls, yet there was a trend for velocity to increase on retesting of controls and to decrease when DBS was switched off.

It has been demonstrated that the UPDRS Part III items most closely associated with VMC performance measures are those related to axial function [56], and this study intriguingly provides a further example of VMC performance relating to axial control in the sense that they both are relatively resistant to STN-DBS.

Changes in affect are an unlikely explanation for the lack of benefit of STN-DBS on VMC as evidenced by the marked deterioration in clinical measures of motor function and the absence of mood changes in the off-stimulation state versus the on-stimulation state.

Our study group comprises patients with advanced PD. This may be particularly relevant because functional disability in the later stages of the disease is often due to non-motor symptoms that do not respond to STN-DBS. Whether STN-DBS would affect VMC performance in patients at earlier stages of the disease is an important question since (a) there is a trend for STN-DBS to be offered to younger patients earlier in the course of the disease [76] and (b) aspects of PD beyond simple motor function are becoming increasingly identified at all disease stages [77], [78]. Another patients’ selection bias is introduced by the a priori selection for positive responders to levodopa/apomorphine treatment, which results from the fact that post-operative improvement in motor symptoms is directly correlated with such response, prior to the operation [79], [80]. A third selection bias for well-preserved cognitive function arises from the requirement for intensive post-operative patient follow-up, as well as the risk of cognitive decline as a direct result of stimulation [81]. Accordingly, it is possible that the range of cognitive changes due to STN-DBS stimulation was narrow in comparison to the motor domain, as seen in the VMC testing results. However, this possibility is not too likely because many studies have demonstrated cognitive decline in early PD patients, especially in the executive domain [82], which are highly relevant to VMC performance [56].

Another possible explanation for underestimating the effect of STN-DBS is that the interval between switching off DBS and starting the second VMC test (minimum of 20 minutes) may not have been long enough to observe all of the ‘off’ effects. However, the magnitude of the change in UPDRS scores clearly demonstrates that clinically subjects did switch into an ‘off’ state. In the present study the mean change in the UPDRS was 55%, which is comparable to that found in the literature after an off period (between 40% [83] and 58% [84] after a 2-hour ‘off’ period, or after an ‘off’ period of one hour 30% reduction at 6 months post-operative and 40% reduction at 3 months post-operative [85]). It has also been shown that on turning off STN-DBS stimulation 90% of the maximal mean group change occurred within 15 minutes for tremor, and 75% of the maximal mean group change occurred between 15 and 30 minutes for bradykinesia [86].

The heterogeneity of the results may largely be explained by the dichotomy between ‘lower’ level motor function and ‘higher’ level motor function. The motor part of the UPDRS is a rating scale for several motor items. These items reflect very basic stereotypical actions which are routinely performed and assessed in clinical situations. Thus only minimal higher level motor planning and cognitive processing is required for successful performance of these movements. On the other hand, visuo-motor performance as tested in this study requires not only the understanding of complex instructions but high-level cognitive processing and visuo-spatial awareness (including transformation of visual representations of hand movements and paths/targets into appropriate motor plans and monitoring of results with corrective feedback loops), motor sequence planning, goal shifting and sensory integration. One interpretational problem is that visuo-motor performance is affected by the patients’ difficulties in movement production. Since we observed a significant clinical improvement in movement production, this is unlikely to be a plausible explanation for the lack of STN-DBS effect on VMC performance. UPDRS motor scores and VMC testing are measuring very different aspects of motor function with the former pertaining more directly to the end motor apparatus. With DBS acting primarily on motor cortex outflow to the STN it is perhaps unsurprising that UPDRS scores improved with DBS stimulation, and VMC testing scores generally did not show a significant change.

Another important aspect of this research relates to the potential clinical applications of VMC testing as a tool that may quantify complex motor function. Certainly in the later stages of disease these aspects are more likely to have a greater impact on disease-related disability than the clinical measures of bradykinesia, tremor and rigidity. By studying patients prospectively with an initial pre-operative assessment that includes visuo-motor function analysis it may be possible to identify certain patient VMC profiles with prognostic value for post-operative functional outcome.

Further studies with larger samples are needed to further investigate which elements of high-level motor function can be improved by STN-DBS or even are at risk of deterioration. Indeed it remains to be determined which aspects of pre-operative functioning should be focused on for the purpose of patient selection for operation. It is possible that certain aspects of high-level motor function, such as visuo-motor function as measured in this study, may have particular value in this context.
